# Dim Flicker: An Endogenous Visual Percept and Its Disease Associations

**DOI:** 10.3390/jcm15020622

**Published:** 2026-01-13

**Authors:** Abdullah Amini, Adam Besic, Avery Freund, Yousif Subhi, Oliver Niels Klefter, Jes Olesen, Jette Lautrup Frederiksen, Michael Larsen

**Affiliations:** 1 Department of Ophthalmology, Rigshospitalet, Valdemar Hansens Vej 1-27, DK-2600 Glostrup, Denmark; adam.besic.01@regionh.dk (A.B.); averygfreund@gmail.com (A.F.); ysubhi@gmail.com (Y.S.); oliver.niels.klefter.01@regionh.dk (O.N.K.); miclar01@regionh.dk (M.L.); 2 Faculty of Health and Medical Science, University of Copenhagen, DK-2100 Copenhagen, Denmark; 3 Department of Clinical Research, University of Southern Denmark, DK-5230 Odense, Denmark; 4 Danish Headache Center, Department of Neurology, Rigshospitalet, DK-2600 Glostrup, Denmark; jes.olesen@regionh.dk; 5 Department of Neurology, Rigshospitalet, DK-2600 Glostrup, Denmark; jette.lautrup.battistini@regionh.dk

**Keywords:** visual illusion, flicker, migraine aura, retinal venous congestion, retinal vein occlusion, central retinal vein occlusion, paracentral acute middle maculopathy, ischemia, retinal hypoperfusion, retinal hemodynamics, optical coherence tomography, central serous chorioretinopathy

## Abstract

**Background/Purpose:** Four patients independently reported episodes of seeing a dimly flickering overlay on an otherwise intact part of their binocular visual field. The aim of the study was to describe the clinical characteristics of this episodic phenomenon, which we call dim flicker. **Methods:** Retrospective chart review and patient evaluation of an animated reference simulation. **Results:** The patients described repeated episodes of a seeing a patch of rhythmically oscillating dim flicker overlaid on a circumscribed patch of their otherwise normal binocular visual field. The flicker was typically seen at low ambient light levels and disappeared in bright light or when one or both eyes were covered. Episodes lasted seconds to minutes. Some flicker patches crossed the vertical midline. The flicker was subjectively experienced as coming from one specific eye. Compared to a 7 Hz flicker simulation, patients reported differences in location, prominence, and frequency, with the latter ranging from 3 to 10 Hz. In three patients, the flicker was sometimes experienced during aerobic exercise and in two patients sometimes when they rose at night in the dark. In one patient, the flicker corresponded to an area of ischemic macular edema secondary to central retinal vein occlusion. There was no headache during or after the flicker. Associated maladies included retinal venous congestion, central serous chorioretinopathy, arterial hypertension, atrial fibrillation, and migraine with visual aura distinctly different from the dim flicker. **Conclusions:** Episodes of seeing an endogenous, rhythmically oscillating transparent overlay within a confined, non-expanding part of an otherwise intact binocular visual field appears to be a distinct nosological entity that can be associated with ocular and systemic vascular disease.

## 1. Introduction

Endogenous visual experiences include seeing retinal vessel shadows [1], circulating leukocytes in perifoveal vessels, phosphenes elicited by the pull of extraocular muscles during voluntary eye movements [2] or by vitreoretinal detachment [3,4], shadows produced by intraocular hemorrhage [5], chromatic halos produced by corneal edema in narrow angle glaucoma [6], and expanding and location-shifting scotomata, sometimes scintillating, in visual migraine aura [7]. Here, we present what appears to be a hitherto undescribed type of episodic rhythmic dim flicker of stationary location and extent, which is seen as a transparent overlay on an otherwise intact part of the binocular visual field.

## 2. Methods

This retrospective review of cases is based upon a study of fundus imaging methodology that recruited volunteer patients from of a medical retina clinic. Patients gave written informed consent, all procedures followed the Helsinki Declaration, and the study was approved by Regional Committee on Health Research Ethics (H-19068888). Patient interviews recorded visual disturbances, when present, with attention to color saturation, contrast, metamorphopsia, sidedness, frequency, time of day, associated events, and other characteristics. Discovery of the dim flicker phenomenon was an unplanned incidental byproduct of the clinical management of cases.

**Figure 1:**
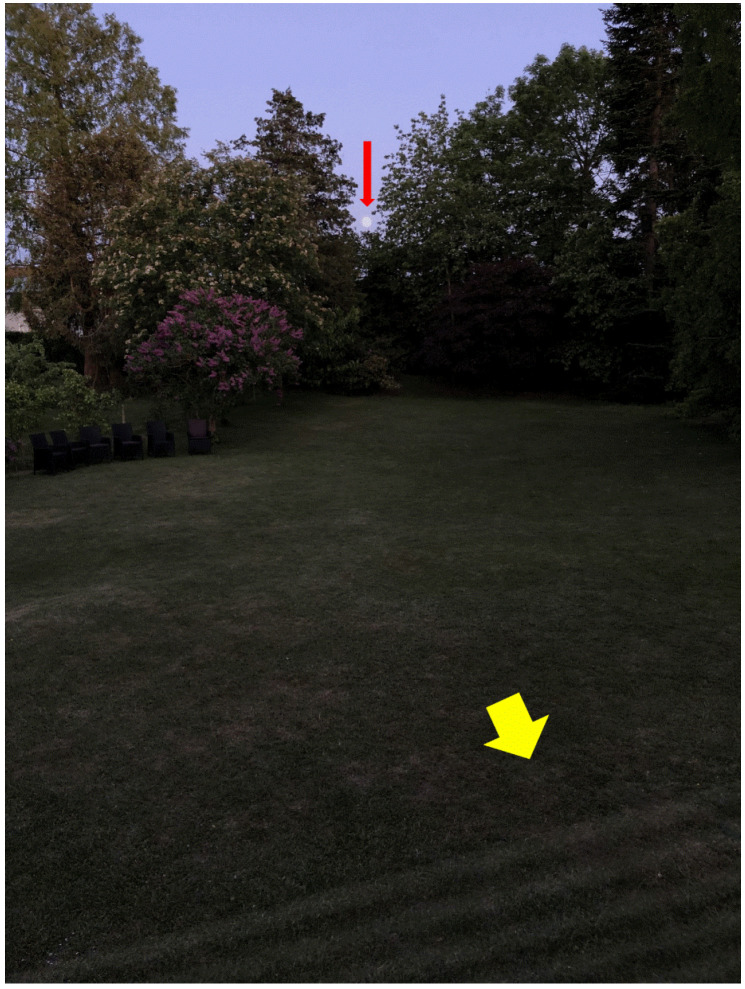
Simulated image of dim flicker produced under the direction of patient 1, who reported intermittently seeing the phenomenon in dim nighttime illumination (moonlight or darker). When fixating on the moon in the top of the image (red arrow), the oblique grating (yellow arrow) in the lower right part of the figure is flickering at a rate of 7 Hz in the animated version (see https://drive.google.com/file/d/1c7r5fX2-gQeV2LYJPSM_JsSGsSFb5NgO/view?usp=sharing (accessed on 20 November 2025)).

The index patient (Table 1; Patient 1) occasionally mentioned seeing flickering in a segment of his visual field and provided a thorough description that prompted the development of an animated simulation (Figure 1; for authentic animated version of dim flicker produced under the direction of patient 1, who reported intermittently seeing the phenomenon in dim nighttime illumination (moonlight or dimmer) see https://drive.google.com/file/d/1c7r5fX2-gQeV2LYJPSM_JsSGsSFb5NgO/view?usp=sharing (accessed on 20 November 2025). When fixating on the moon at the simulation, the flicker resembles the oblique grating at the right lower part of the simulation, which flickers at a rate of 7 Hz). The simulation was used for comparison when interviewing subsequent patients who described similar visual experiences. Patients were asked to compare the location, extent, rate, transparency, and background contrast of their flicker with the simulation. They were also asked about time of their first event, how often it recurred, and the time of day and duration of events, as well as precipitating or modifying factors. Medical history and uses of medications were also recorded (Table 1).

An electronic search on PubMed, Google Scholar, and SCOPUS was conducted using terms that describe established endogenous visual experiences. The keywords “entoptic phenomenon”, “entoptic flicker”, “entoptic pulsating”, and “dim flicker” achieved 353 hits, but did not lead to any description of a rhythmically pulsating endogenous visual experience that was fully compatible with dim flicker. Two patients in one migraine aura study provided short unstructured verbal descriptions that are partially compatible [8], but do not cover all essential dim flicker characteristics. The literature search was limited by lack of detail in the terminology used to classify of visual disturbances in systems such as the ICD-10. The literature search was completed on 14 July 2025.

## 3. Results

### 3.1. Case 1

A 48-year-old man presented for a routine eye examination (Table 1). His past medical history included migraine with classic visual aura, arterial hypertension, atrial fibrillation, pacemaker implantation, and medication with metoprolol, losartan, amlodipine, and apixaban. Best-corrected visual acuity (BCVA) was 1.25 Snellen in both eyes, refraction −0.5 spherical in both eyes, and applanation tonometry 12 mmHg in both eyes. Automated perimetry was well within normal limits. His visual migraine aura was mostly paracentral and negative, sometimes with scintillations, but never transparent. The aura was always expanding, never with a rhythmic flicker, and mostly without subsequent headache.

Two years earlier, the patient first noticed a flickering overlay in the inferior periphery of his binocular visual field while cross-country skiing at night on a dimly illuminated path. The flicker was centered to the left of the vertical midline and extended into the lower right quadrant. Vision was otherwise normal, within the flickering area and elsewhere. After approximately 5 min, the flicker waned, and the patient continued skiing without headache, atrial fibrillation, pain, or other physiological abnormalities. The patient subsequently noticed the same flicker phenomenon at long intervals, mostly when standing during nightly micturition in faintly lit surroundings. It faded when he covered his eyes, so that he was unable to determine if it was binocular or monocular. Flicker episodes were not associated with migraine aura or headache. A digital simulation of the patient’s description (Figure 1) was rated as fitting, albeit with more contrast than the patient’s flicker. The condition has remained unchanged for 8 years with flicker episodes weeks or months apart.

### 3.2. Case 2

A 52-year-old woman was referred with blurred and distorted vision in her left eye, BCVA 1.0 Snellen in both eyes and applanation tonometry 14 mmHg in both eyes (Table 1). The patient also reported years of spontaneous episodes of transparent flicker in her left eye, visible with eyes opened or closed. Tangent screen visual fields were normal. There were multiple minor extrafoveal pigment epithelium detachments in both eyes and a serous detachment in her left fovea. She had been treated for half a year for coughing and shortness of breath using oral prednisolone and salbutamol inhalation, until the working diagnosis was changed from asthma to sequelae of pulmonary embolism. Pulmonary artery pressure was normal and arterial blood pressure 148/97 mmHg. The patient was switched to formoterol inhalation, medroxyprogesterone, and warfarin. She underwent verteporfin–photodynamic treatment for central serous chorioretinopathy in her left eye, after which the foveal detachment resolved and metamorphopsia and dim flicker in the left eye disappeared.

Two years later, the patient returned with intermittent flicker, mostly in the upper quadrants of the right eye, resembling sunshine reflecting on rippled water, comparable to but fainter than the simulation (Figure 1). Flicker appeared every one to four days, sometimes after strenuous exercise or during nocturnal micturition in dim light. There was no associated headache. The flicker disappeared when room lights were turned on. Periodically, and symptomatically distinct from the dim flicker episodes, the patient had visual migraine auras with a non-flickering homonymous scotoma of varying sidedness followed by migraine headache.

### 3.3. Case 3

A 69-year-old woman presented with sudden vision loss in her left eye (Table 1). She was known with hypercholesterolemia, hypothyroidism, gastric reflux, and asthma and treated using clopidogrel, rosuvastatin, levothyroxine, salbutamol, salmeterol, fluticasone, and pantoprazole. Her blurred vision was accompanied by short episodes of semitransparent flicker with additional clouding and color desaturation in the central part of the visual field of her left eye. When seen 12 h after onset, BCVA was 0.8/0.3 Snellen, refraction +2.0 spherical in both eyes, and applanation tonometry 15 mmHg. Her right eye was normal, whereas the left eye had profusely dilated retinal veins and widespread perivenular ischemic hyperreflectivity of the inner and middle retinal layers consistent with ischemic central retinal vein occlusion with cilioretinal artery pseudoocclusion. There was no cystoid macular edema. Normal sedimentation rate, C-reactive protein, and clinical examination were taken to rule out temporal arteritis. Hemoglobin was 10.6 (7.3–9.5 mmol/L), leukocytes 11.3 (3.5 × 10^9^/L–8.8 × 10^9^/L), thrombocytes 416 (145–390), monocytes 0.94 (0.20–0.80), and neutrophils 7.17 (1.6–5.9), all of which were normal when checked again half a year later. The cause of polycythemia was not identified. Ultrasonography of the carotid arteries was unremarkable. Cerebral magnetic resonance imaging showed an old infarct unrelated to the visual pathways. Additional flicker episodes occurred in the next days. After 10 months, BCVA had spontaneously recovered to 1.0 Snellen in both eyes, but a paracentral relative scotoma was found corresponding to the core of the previously ischemic, now atrophic paracentral area of the macula.

The patient described the flicker simulation (Figure 1) as a good match to her own in terms of amplitude and transparency, but her flicker was slower, about 3 Hz, and the acute phase of her visual loss was dominated by loss of color combined with a lesser element of flicker within a single central vaguely outlined part of the visual field of her left eye. The flicker episodes occurred in bouts lasting no more than 1 min without any identifiable precipitating factor.

### 3.4. Case 4

A 46-year-old man reported occasional blurred vision and episodes of flickering in his right inferior hemifield during and after habitual 10 km jogging exercises (Table 1). The flicker was particularly frequent four weeks before his first visit, when he was found to have central retinal vein occlusion without macular edema in his right eye with hemorrhages in the inferior hemifield. Nine years before, he had experienced dim flicker in his left eye in relation to having upper hemicentral retinal vein occlusion without macular edema. Both venous congestion and flicker in his left eye had disappeared spontaneously with the development of collateral venous drainage on the optic disk. His BCVA was 1.0 Snellen in both eyes, applanation tonometry 18/20 mmHg, automated perimetry normal in the right eye and marginally subnormal in the lower hemifield of his left eye. The patient did not use any medication. Compared to the simulation (Figure 1), the flicker in his right eye was faster, closer to the center of the visual field, of lower amplitude than the simulation (Figure 1), and spreading homogeneously over a large, uniform patch without striping. In addition to the jogging-related episodes, the patient occasionally saw flicker in his right eye when waking from sleep in the recumbent position, before and for up to 30 min after opening his eyes. Retinal venous congestion and hemorrhage in his right eye waxed and waned and had resolved after half a year. He continued to experience flicker episodes in his right eye, mostly while jogging.

## 4. Discussion

Four patients reported episodes of seeing a faint transparent flickering overlay within a single patch of their binocular visual field. Most observations of dim flicker were made in low ambient luminance and the flicker was quenched or dominated by bright light. The flickering patch did not expand or contract during episodes and was not followed by headache. The flicker pattern was multiple parallel lines in two patients (Figure 1) and a single uniform patch in the other two. Flicker rates ranged from 3 to 10 Hz and were constant for individual patients during episodes and from episode to episode, which lasted seconds to minutes and were hours to months apart.

All patients described their dim flicker as coming from a specific eye. This concurred with the presence of retinal venous congestion in two cases, but it is uncertain whether this impression was triggered by the unilateral blurred vision these patients had between their flicker events.

Flicker episodes were associated with waking from sleep (three patients), prolonged aerobic exercise (three patients), retinal venous congestion (two patients), and polycythemia (one patient). Alleviating factors included bright ambient light and reduction in venous congestion (two patients). Two had cardiovascular disease but no obvious link between these conditions and the flicker.

Unlike visual migraine aura, dim flicker did not move or expand during episodes [7] and was not combined with a scotoma [9] or followed by headache [10]. In contrast to visual migraine aura, the dim flicker had a regular beat and did not scintillate. Two dim flicker patients who had migraine with visual aura reported that their dim flicker was distinctly different from their auras. Dim flicker spanning across the vertical midline in two cases also suggests an origin anterior to the optic chiasm. No patient had a history of epilepsy, substance abuse, multiple sclerosis [11], or other potential cerebral cause of visual disturbances.

Endogenous flickering may theoretically be induced by external pressure on the eye, retinal traction, vitreous hemorrhage, occipital epilepsy, or transient cerebral ischemia [4], none of which apply in our cases.

The type of visual experience described by our patients has not, to the best of our knowledge, previously been described as a distinct entity. It may have been interpreted and categorized, however, as a form of migraine aura [12]. A study of migraine where patients compared their auras to a selection of static images includes an example entitled “Like looking through heat waves, water or oil” and described by one patient as “Experiencing flicker in one eye. May feel like you have tic on your eye. It flutters quickly, and only the bright colors become prominent” and by another patient as “I constantly see small lines that sort of sprinkle down”, suggesting that the patients may actually have had dim flicker [8].

Visual migraine aura is the most common aura, reported by more than 90% of migraine patients [7]. Its cortical origin is well documented [12,13]. The International Classification of Headache Disorders, 3rd edition, includes two types of auras with a visual percept: visual and retinal aura [13], (Table 2 and Table 3). The former has its origin in the visual cortex [14] and the latter, by definition, in the retina [15]. The terminology is ambiguous, however, because it makes no distinction between positive sensory disturbances, i.e., the illusion of seeing something (scintillations) and negative sensory disturbances with local absence of vision producing a blank scotoma, often filled in by interpolation from the intact surrounding visual field. Positive disturbance should appear the same in both eyes, whether they are produced by the visual cortex or by one of the two retinae, such as phosphenes produced by indenting the retina through the eyelid, and they should be seen with eyes open as well as eyes closed. Thus, a luminous percept generated by the visual cortex cannot be distinguished from one generated by the retina [16]. On the contrary, the origin of negative disturbances should be identifiable, since the scotoma will be binocular and homonymous in cortical lesions, but uniocular in retinal lesions, and in both cases visible only with eyes open. These complex interactions between anatomy and function have not always been fully addressed in the literature and eye examination data collected during purported retinal migraine attacks are scarce. Nevertheless, qualified and well-documented observation has been made of recurrent acute unilateral retinal vasoconstriction with transient blindness in the affected eye [17]. It is debatable, however, whether these cases are properly described by the term retinal migraine, in the sense that they lack the gradual spreading and subsequent headache required by the International Headache Society classification of migraine [13] (Table 2).

The present study does not permit the identification of a unifying underlying mechanism behind episodic dim flicker, but the associated factors of retinal vein occlusion, recumbency during sleep, polycythemia, and strenuous exercise with a potential for dehydration suggest that abnormal retinal perfusion may be a shared feature.

We define dim flicker as an episodic, rhythmically flickering overlay on an otherwise intact visual field. All cases we know of so far were monosymptomatic and recurrent. Awareness of dim flicker may promote the diagnosis of underlying disease and should lead to a thorough examination of the eye and of visual function.

## Figures and Tables

**Table 1:** Endogenous flicker characteristics.

	Patient 1	Patient 2	Patient 3	Patient 4
Age, sex	48 y, male	52 y, female	69, female	46 y, male
Visual field location of flicker	Periphery of inferior quadrants, right eye	Upper visual field, waves in motion, mostly right eye, in left eye never after PDT for CSC	Left eye, straight ahead,	Inferior quadrants, right eye
Best-corrected visual acuity OD/OS	1.25/1.25	1.0/1.0	0.9/0.4	1.0/1.0
Flicker rate [Hz]	7	7	3	10
Pattern	Oblique grating	Oblique ripples	Grating	Uniform patch
Visibility	Faint but distinct	Faint but distinct	Distinct	Faint but distinct
Transparency	High	High	High	High
Duration	Minutes	Minutes	Days	Minutes
Timing	Spontaneous, episodically, approx. 10 times per year	Spontaneous, up to hours	Left eye suddenly dark, after 1 h, finger counting but no colors, after 2 days near-normal colors, no flicker	Spontaneously, during and after jogging, after lying down
Ambient luminosity	Dim nocturnal after awakening and standing	Dim nocturnal and sometimes in daylight	Any	Any
Stimulating factors	After waking from sleep and while exercising	After strenuous exercise and in dim light	During event with retinal vein occlusion	Onset of flicker while jogging and exacerbation of events during episodes of retinal venous congestion
Alleviating factors	Ambient light	Ambient light	Faded after regression of retinal vein occlusion.	Faded with spontaneous regression of venous congestion.
Automated perimetry, OD/OS, mean deviation (−2,2)	1.1 dB/−0.5 dB (normal)	2.0 dB/4.0 dB (inferior to the fovea)	7.2 dB/2.7 dB (nasal quadrants)	3.4 dB/1.9 dB (irregular)
Medications	Metoprolol, losartan, apixaban, amlodipine	Dabigatran, formoterol	Pravastatin, levothyroxine, salbutamol, fluticasone	None
Past medical history	Atrial fibrillation, arterial hypertension, migraine with cortical and retinal visual aura	CSC OU with cessation of flicker OS after PDT, persisting OD. Pulmonary embolism. Occasional visual aura without migraine, non-scintillating, no flicker	Central retinal vein congestion with branch retinal artery hypoperfusion, cataract OU, hypercholesterolemia, hypothyroidism, asthma	CRVO OD, earlier hemi-CRVO OS. Left-side ophthalmic nerve herpes zoster, anterior uveitis

CSC: Central serous chorioretinopathy. OD, OS, OU: Right eye, left eye, both eyes. CRVO: Central retinal vein occlusion. PDT: Verteporfin photodynamic therapy. dB: Decibel.

**Table 2:** Diagnostic criteria for migraine with aura (ICHD-3) [13].

A. At least two attacks fulfilling criteria B and C
B. One or more of the following fully reversible aura symptoms:
1. Visual. 2. Sensory. 3. Speech and/or language. 4. Motor. 5. Brainstem. 6. Retinal.
C. At least three of the following six characteristics:
1. At least one aura symptom spreads gradually over ≥5 min
2. Two or more aura symptoms occur in succession
3. Each individual aura symptom lasts 5–60 min
4. At least one aura symptom is unilateral *
5. At least one aura symptom is positive (scintillations or pins and needles **)
6. The aura is accompanied, or followed within 60 min, by headache
D. Not better accounted for by another ICHD-3 diagnosis and transitory ischemic attack has been excluded

(*) Unilateral means on one side of the midline of the body or the midline of the binocular visual field. (**) Pins and needles refer to the sensation of a type of pain without any specific relation to the eye or vision.

**Table 3:** Diagnostic criteria for retinal migraine (ICHD-3) [13].

Description: Repeated attacks of monocular visual disturbance, including scintillations, scotomata, or blindness, associated with migraine headache.
Diagnostic criteria:
A. Attacks fulfilling criteria for migraine with aura (see above) and criterion B below:
B. Aura characterized by both of the following:
1. Fully reversible, monocular, positive and/or negative visual phenomena (e.g., scintillations, scotomata or blindness) confirmed during an attack by either or both of the following:
a. Clinical visual field examination.
b. The patient’s drawing of a monocular field defect (made after clear instruction).
2. At least two of the following:
a. Spreading gradually over ≥5 min.
b. Symptoms last 5–60 min.
c. Accompanied or followed within 60 min by headache.
C. Not better accounted for by another ICHD-3 diagnosis and other causes of amaurosis fugax have been excluded.

## Data Availability

All data generated or analyzed during this study are included in this article. Further enquiries can be directed to the corresponding author.

## References

[1] Sharpe C.R. (1972). The visibility and fading of thin lines visualized by their controlled movement across the retina. J. Physiol..

[2] Tehovnik E.J., Slocum W.M., Carvey C.E., Schiller P.H. (2005). Phosphene induction and the generation of saccadic eye movements by striate cortex. J. Neurophysiol..

[3] Nixon T.R.W., Davie R.L., Snead M.P. (2024). Posterior vitreous detachment and retinal tear—A prospective study of community referrals. Eye.

[4] Gishti O., van den Nieuwenhof R., Verhoekx J., van Overdam K. (2019). Symptoms related to posterior vitreous detachment and the risk of developing retinal tears: A systematic review. Acta Ophthalmol..

[5] Kanski J.J. (1975). Complications of acute posterior vitreous detachment. Am. J. Ophthalmol..

[6] Nüßle S., Reinhard T., Lübke J. (2021). Acute closed-angle glaucoma. Dtsch. Arztebl. Int..

[7] Thomsen A.V., Ashina H., Al-Khazali H.M., Rose K., Christensen R.H., Amin F.M., Ashina M. (2024). Clinical features of migraine with aura: A REFORM study. J. Headache Pain.

[8] Viana M., Hougaard A., Tronvik E., Winnberg I.G., Ambrosini A., Perrotta A., Do T.P., Al-Karagholi M.A.-M., Fominykh M., Sihabdeen S. (2024). Visual migraine aura iconography: A multicentre, cross-sectional study of individuals with migraine with aura. Cephalalgia.

[9] Schankin C.J., Viana M., Goadsby P.J. (2017). Persistent and Repetitive Visual Disturbances in Migraine: A Review. Headache.

[10] Shah D.R., Dilwali S., Friedman D.I. (2018). Current Aura Without Headache. Curr. Pain. Headache Rep..

[11] Frohman T.C., Davis S.L., Beh S., Greenberg B.M., Remington G., Frohman E.M. (2013). Uhthoff’s phenomena in MS--clinical features and pathophysiology. Nat. Rev. Neurol..

[12] Hill D.L., Daroff R.B., Ducros A., Newman N.J., Biousse V. (2007). Most Cases Labeled as “Retinal Migraine” Are Not Migraine. J. Neuro-Ophthalmol..

[13] Olesen J. (2018). Headache Classification Committee of the International Headache Society (IHS) The International Classification of Headache Disorders, 3rd edition. Cephalalgia.

[14] Hadjikhani N., Sanchez Del Rio M., Wu O., Schwartz D., Bakker D., Fischl B., Kwong K.K., Cutrer F.M., Rosen B.R., Tootell R.B.H. (2001). Mechanisms of migraine aura revealed by functional MRI in human visual cortex. Proc. Natl. Acad. Sci. USA.

[15] Carroll D. (1970). Retinal migraine. Headache J. Head Face Pain.

[16] Evans I.D., Palmisano S., Croft R.J. (2021). Retinal and Cortical Contributions to Phosphenes During Transcranial Electrical Current Stimulation. Bioelectromagnetics.

[17] Doyle E., Vote B.J., Cosswell A.G. (2004). Retinal migraine: Caught in the act. Br. J. Ophthalmol..

